# Gastrodin promotes the regeneration of peripheral nerves by regulating miR-497/BDNF axis

**DOI:** 10.1186/s12906-021-03483-z

**Published:** 2022-02-18

**Authors:** Li Yongguang, Wang Xiaowei, Yan Huichao, Zhang Yanxiang

**Affiliations:** Department of Orthopedics, Hubei No.3 People’s Hospital of Jianghan University, 26 Zhongshan Avenue, Qiaokou District, Wuhan, 430000 Hubei China

**Keywords:** Gastrodin, miR-497, Brain-derived neurotrophic factor, Peripheral nerve injury

## Abstract

**Background:**

Gastrodin (GAS), is a kind of phenolic compound extracted from the traditional Chinese herbal medicine Gastrodia elata Blume (GEB). This study was aimed at probing into the protective effect of GAS on peripheral nerve injury (PNI) and the underlying mechanism.

**Methods:**

A rat model with PNI was established, followed by intraperitoneal injection of GAS (20 mg/kg/day). Sciatic nerve function index (SFI) was used to analyze the function of sciatic nerve. The amplitude and latency of compound muscle action potential (CMAP) were examined by electrophysiology. Schwann cells (SCs) were isolated from fetal rats and treated with GAS 200 μg/mL, and H_2_O_2_-induced model of oxidative stress injury was established. EdU and Transwell assays were adopted to detect the viability and migration of SCs. Dual-luciferase reporter gene assays were applied to verify the binding site between miR-497 and brain-derived neurotrophic factor (BDNF) 3’UTR. MiR-497 expression was probed by quantitative real-time polymerase chain reaction (qRT-PCR). BDNF, neurofilament-200 (NF-200) and myelin basic protein (MBP) expression levels were detected by Western blotting. Malondialdehyde (MDA) content, superoxide dismutase (SOD) activity, glutathione content (GSH) and catalase (CAT) activity in SCs were also measured.

**Results:**

GAS treatment could significantly increase the SFI and amplitude of CMAP, shorten the refractory period, and ameliorate muscle atrophy of the rats with PNI. GAS treatment could markedly restrain miR-497 expression and increase the expression levels of BDNF, NF-200 and MBP in SCs. BDNF was confirmed as the target of miR-497 and BDNF overexpression could reverse the impacts of miR-497 overexpression on the proliferation, migration, and oxidative stress response of SCs.

**Conclusions:**

GAS promotes the recovery of PNI via modulating miR-497 / BDNF axis and inhibiting oxidative stress.

**Graphical abstract:**

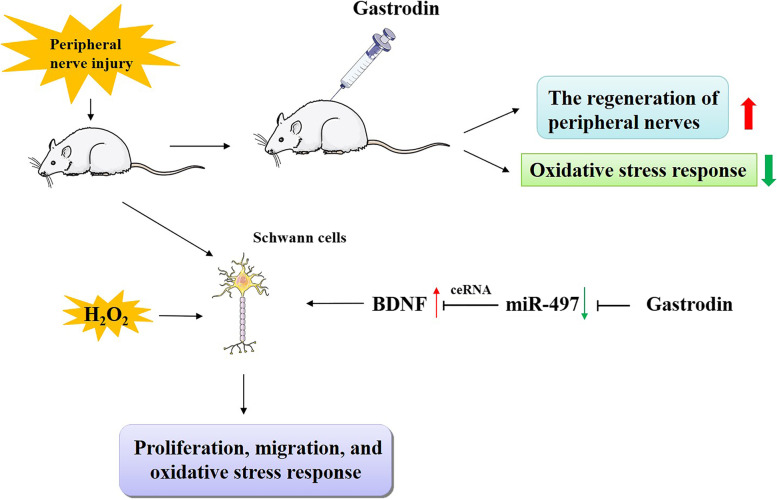

**Supplementary Information:**

The online version contains supplementary material available at 10.1186/s12906-021-03483-z.

## Background

Peripheral nerve injury (PNI) is a prevailing clinical problem leading to the disability of the patients and socioeconomic burden [1]. PNI is caused by diverse factors, such as rupture, stretch, compression and ischemia, fracture and iatrogenic injury [2]. Besides, PNI is often accompanied by inflammatory reaction, oxidative stress, and nutritional deficiency. Unlike central nervous system (CNS), peripheral nervous system (PNS) has the ability to regenerate itself after injury [3]. Schwann cell (SC), is the main glial cell in PNS, and it is pivotal in regulating the regeneration of peripheral nerves [4, 5]. Reportedly, SCs can produce and secrete nerve growth factor (NGF) and brain-derived neurotrophic factor (BDNF), which are implicated in the post-PNI growth and regeneration processes of peripheral nerve [6–10]. Promoting the multiplication and migration of SCs can improve the regeneration of peripheral nerves [11, 12]. Although PNS possesses certain regeneration capabilities, in clinical practice, the structural and functional recovery after PNI is far from satisfactory [13]. Therefore, it is urgent to develop novel and effective drugs to enhance nerve regeneration and to improve the treatment of PNI.

Reportedly, microRNA (miRNA) plays a regulatory role in neurodevelopment and neuron degeneration, and miRNAs are considered as novel therapy targets for neurological injury [14, 15]. For example, miR-21 inhibition modulates the epidermal growth factor receptor pathway, reduces excessive activation of astrocytes in a model of optic nerve injury, and promotes axon regeneration in a rat model [16]. MiR-497 is known as a tumor suppressor in diverse human cancers [17, 18]. Moreover, it is also reported that miR-497 takes part in regulating the death and survival of neurons [19, 20]. Notably, miR-497 inhibition can ameliorate cerebral ischemic injury via enhancing autophagy [21]. However, the function of miR-497 in peripheral nerve regeneration is not well-studied.

Gastrodia elata Blume (GEB), a well-known Chinese herbal medicine, is widely adopted to treat migraine, dizziness, tetanus, epilepsy, stroke, amnesia and other diseases in China and other Asian countries [22]. Gastrodin (GAS), a phenolic glycoside, is the main bioactive component extracted from GEB, and it is reported to have certain beneficial effects on diverse neurological diseases; it can regulate oxidative stress, neuroinflammation, microglial activation, mitochondrial dysfunction and so on [23, 24]. For example, GAS restrains inflammation, oxidative stress, and apoptosis in the hippocampus, which exhibits neuroprotective effects against cognitive dysfunction in diabetes mellitus rats [25]. GAS is one of the main ingredients in Da Chuanxiong Formula (DCXF). It is found that DCXF treatment can decrease blood-brain barrier (BBB) leakage and brain edema, reduce neuron loss and microglia activation, which can ameliorate traumatic brain injury-induced brain damage in rats [26]. Furthermore, it is reported that GAS modulates SCs proliferation through repressing ERK1/2 phosphorylation and activating Akt phosphorylation, implying its potential to treat PNI [27]. Nevertheless, the protective effect of GAS on PNI still awaits more elucidation.

This study was designed to investigate the influence of GAS on nerve regeneration and functional recovery in a rat model with PNI in vivo. Besides, with in vitro experiments, we also delved into the function of GAS on SCs’ viability and migration, and its regulatory function on miR-497 and BDNF and demonstrated that GAS promoted the viability and migration of SCs via regulating the miR-497 / BDNF axis, attenuated oxidative stress response, and thus exerted neuroprotective effects on PNI.

## Methods and materials

### Rats model

In animal experiments, all the procedures were carried out in comply with the National Institute of Health Guidelines for Care and Use of Laboratory Animals in Biomedical Research, and endorsed by the Research Ethics Committee of Hubei Third People’s Hospital of Jianghan University. Generally, male Sprague-Dawley (SD) rats (6-week-old, 180–200 g) (Institute of Model Animal, Wuhan University, Wuhan, China) were raised at room temperature, with a light-dark cycle of 12 h/12 h and adequate food and water. The rats were randomly divided into four groups (*n* = 5 in each group): sham group, PNI group, PNI + Veh group (injection of normal saline) and PNI + GAS treatment group [intraperitoneal injection of GAS (purity ≥98%, Sigma-Aldrich, Louis, MO, USA), 20 mg/kg/day for 2 consecutive weeks after the surgery]. To induce PNI, rats were anaesthetized by intraperitoneal injection of 3% pentobarbital sodium (50 mg/kg) after weighing, The animals were with stable breathing, muscle relaxation and no reaction to acupuncture on the plantar, which indicated that the anesthesia was successful [28]. Next, the right sciatic nerve was exposed and then clamped with hemostatic forceps for 3 times, 10 s for each time. After GAS treatment and functional experiments, the rats were euthanized using a chamber pre-filled with 70% carbon dioxide (CO_2_) gas, which caused the loss of consciousness and controlled the pain. After that, the proximal sciatic nerve (about 0.3 cm in length) was obtained for extracting the protein and RNA, and gastrocnemius muscle was also obtained.

### Sciatic nerve function index (SFI)

SFI was measured on 1, 4, 7, and 14 d after surgery. Briefly, the hind paws of the rats were specifically marked with black ink before the rats were subsequently released to walk along a corridor (6 × 60 cm^2^) covered with white paper. Notably, according to the footprints of rats on white paper, SFI was calculated [29]. SFI of − 100 and SFI of 0 indicated the complete loss of function and normal function, respectively.

### Electrophysiological analysis

Compound muscle action potential (CMAP) of the rats was determined to show the number and functional status of excitable cells. Double-blinded experiments were performed by a well-trained experimenter who was not involved in any previous animal grouping and treatment. Two weeks after the surgery, the sciatic nerves of the rats were exposed and the site of nerve repair was accordingly identified under an operating microscope. Next, a rubber dam was adopted to isolate the nerve repair site from the surrounding muscles. Then a bipolar electrode was placed in 10 mm proximal to the repair site of the sciatic nerve for electrical stimulation using an electromyography set (E-Wave, Science Beam, Iran). Ultimately, the provoked CMAP amplitude and CMAP latency on gastrocnemius muscle were recorded.

### Measurement of muscle mass

The gastrocnemius muscles of both the intact and the injured side were dissected and removed from the euthanized rats with ophthalmic scissors and ophthalmic forceps, and immediately weighed. The muscle mass ratio of each rat was calculated: muscle mass ratio = (muscle mass of the injured side)/(muscle mass of the control side) × 100%.

### Cell culture and transfection

The primary SCs were isolated from the sciatic nerve of newborn 1-day-old SD rats with the method described in the previous report [30]. Rat SCs (RSC96 cells) and human embryo kidney epithelial cells (HEK293T) were available from the American Type Culture Collection. The cells were cultured in Dulbecco’s Modified Eagle’s Medium (Hyclone, Logan, UT, USA) with 10% fetal bovine serum (FBS; Hyclone, Logan, UT, USA). Additionally, 5 μM forskolin and 4.1 μg/ml insulin (Sigma, St Louis, MO, USA) were used to stimulate the SCs to grow. MiR-497 mimic, negative control mimic (NC mimic), pcDNA3.1-BDNF (pc-BDNF) or empty pcDNA3.1 vector (pc-NC) were synthesized and provided by GenePharma Co. (Shanghai, China). The above plasmids and oligonucleotides were transfected into SCs with Lipofectamine RNAiMAX transfection reagent (Invitrogen, Carlsbad, CA, USA).

GAS (purity ≥98%, Sigma-Aldrich, Louis, MO, USA) was dissolved in DMSO (Sigma-Aldrich, Louis, MO, USA) and then added in the medium, and the cells were cultured with the working concentration of 200 μg/mL. 24 h later, SCs were cultured in the medium containing 100 μmol/L H_2_O_2_ solution for 4 h to simulate oxidative stress-induced injury.

### Quantitative real-time polymerase chain reaction (qRT-PCR)

To detect miR-497 and BDNF expressions, total RNA was subsequently extracted with TRIzol reagent (Invitrogen, Carlsbad, CA, USA) from the SCs or sciatic nerve. Subsequently, Prime-Script RT reagent Kit (TaKaRa, Dalian, China) was used to reversely transcribe RNA into cDNA. After that, qRT-PCR was performed on the Applied Biosystems StepOne real-time PCR system (Applied Biosystems, Foster City, CA, USA) with SYBR Premix Ex Taq (TaKaRa, Dalian, China). The sequences of the primers were shown as follows: miR-497: F: 5′-AGCGAAGTTTTGAGCCGATCGGGC-3′; R: 5′-GCCGTGAGTCAGAGGTGGT-3′; BDNF: F: 5′-CAGGGGCATAGACAAAAG-3′; R: 5′-CTTCCCCTTTTAATGGTC-3′; U6: F: 5′-GCGCGTCGTGAAGCGTTC-3′; R: 5′-GTGCAGGGTCCGAGG-3′; GAPDH primers: F: 5′-GGAGCGAGATCCCTCCAAAAT-3′; R: 5′-GGCT GTTGTCATACTTCTCATGG-3′. GAPDH and U6 served as the endogenous controls. The relative expressions of the genes were calculated by 2^-ΔΔCt^ method.

### Western blotting assay

The injured sciatic nerve samples and SCs were lysed in RIPA buffer (Beyotime, Shanghai, China) on ice for 30 min to extract the total proteins, which were subsequently quantified by BCA protein quantification kit (Beyotime, Shanghai, China). Following this, the protein samples (20 μg / per lane) were separated with SDS-PAGE and then transferred onto PVDF membranes (Millipore, Bedford, MA, USA). Afterwards, the proteins on the membranes were blocked with 5% skimmed milk at room temperature for 1 h, and the membranes were then interacted with primary antibodies including anti-BDNF (Abcam, Shanghai, China; 1: 500; ab108319), anti-myelin basic protein (MBP) (Abcam; 1: 500; ab7349), anti-neurofilament-200 (NF-200) (Sigma-Aldrich, Louis, MO, USA; 1: 500; N4142), and anti-β-actin (Abcam; 1: 2000; ab8227) at 4 °C for 12 h, respectively. Next, the membranes were washed twice with TBST, and incubated with the corresponding HRP-conjugated secondary antibody (Proteintech, Wuhan, China) at ambient temperature for 1 h. Ultimately, the protein bands on the membranes were developed with ECL kit (Millipore, Bedford, MA, USA), with β-actin as the endogenous control.

### Cell viability assay

The viability of SCs was measured by EdU kit (Ribobio, Guangzhou, China). SCs were transferred into a 96-well plate coated with polylysine (1 × 10^4^ cells / well). Subsequently, the SCs were cultured for 12 h, and then incubated with 50 μmol/L EdU solution for 12 h and then fixed with 4% paraformaldehyde. After that, the cells were stained with Apollo fluorescent staining reaction solution for 30 min, and then DAPI staining solution was loaded to mark the nuclei for 30 min. After that, the cells were observed and counted under a fluorescence microscope (Olympus, Tokyo, Japan). The ratio of the total number of EdU positive cells (red nuclei) to the total number of cells (blue nuclei) indicated the proliferation/viability of SCs.

### Transwell assay

The migration ability of SCs was measured employing Transwell inserts with 8 μm pore size (Corning, NY, USA). The filter of the each Transwell insert was coated with 10 μg/mL fibronectin (Beyotime, Shanghai, China). After that, SCs were inoculated into the top chamber (5 × 10^4^ cells per well), and meanwhile, 600 μL of complete medium containing 20% FBS was injected into the lower chamber. After 24 h of culture, the SCs in the upper chamber were immediately wiped off with cotton swabs. Next, SCs which moved to the lower chamber were stained with 0.5% crystal violet solution for 20 min. Finally, and the migrated SCs were observed and counted with a microscope (Olympus, Tokyo, Japan).

### Luciferase reporter gene assay

Briefly, BDNF 3′-UTR sequence was amplified by PCR and then subcloned into the luciferase reporter vector (Promega, Madison, WI, USA) to construct luciferase reporter plasmids containing wild-type BDNF (pmirGLO-BDNF-WT) or mutant BDNF (pmirGLO-BDNF-MUT). BDNF-WT or BDNF-MUT plasmids and miR-497 mimic or NC mimic were subsequently co-transfected into HEK293T cells by Lipofectamine RNAiMAX transfection reagent (Invitrogen, Carlsbad, CA, USA). 48 h later, Dual-Luciferase Reporter Assay System (Promega, Madison, WI, USA) was adopted to detect the luciferase activity of each group.

### Oxidative stress assessment

The malondialdehyde (MDA) level in nerve tissues and SCs of each group was measured with MDA detection kit (Solarbio, Beijing, China) to evaluate lipid peroxidation by colorimetry. The activities of superoxide dismutase (SOD) and catalase (CAT) of nerve tissues and SCs in each group were detected to evaluate their antioxidant enzyme activities, with corresponding detection kits (Solarbio, Beijing, China) using enzyme-linked immunosorbent assay method. The content of glutathione (GSH) in nerve tissues and SCs of each group was also investigated to indicate the oxidative stress with GSH detection kit (Amersham Biosciences, USA) by colorimetry.

### Statistical analysis

The statistical analysis was performed with SPSS software (SPSS Inc., Chicago, IL, USA) (version 17.0). Generally, all experiments were performed in triplicate and repeated at least three times, and all data were expressed as mean ± standard deviation. Data normality was first confirmed by the Kolmogorov-Smirnov test. For the data which were normally distributed, an unpaired or paired t-test was accordingly adopted to compare the data between 2 groups. Besides, comparisons among ≥3 groups were conducted with one-way ANOVA followed by Tukey’s post hoc analysis. For the data which were skewed distributed, comparisons between 2 groups were accordingly performed by a paired sample Wilcoxon signed-rank test. Statistically, *P* < 0.05 is significant. At last, GraphPad Prism 8 (GraphPad Software, Inc., San Diego, CA, USA) was adopted for drawing the graphs.

## Results

### GAS promotes the repair of PNI

**Fig. 1:**
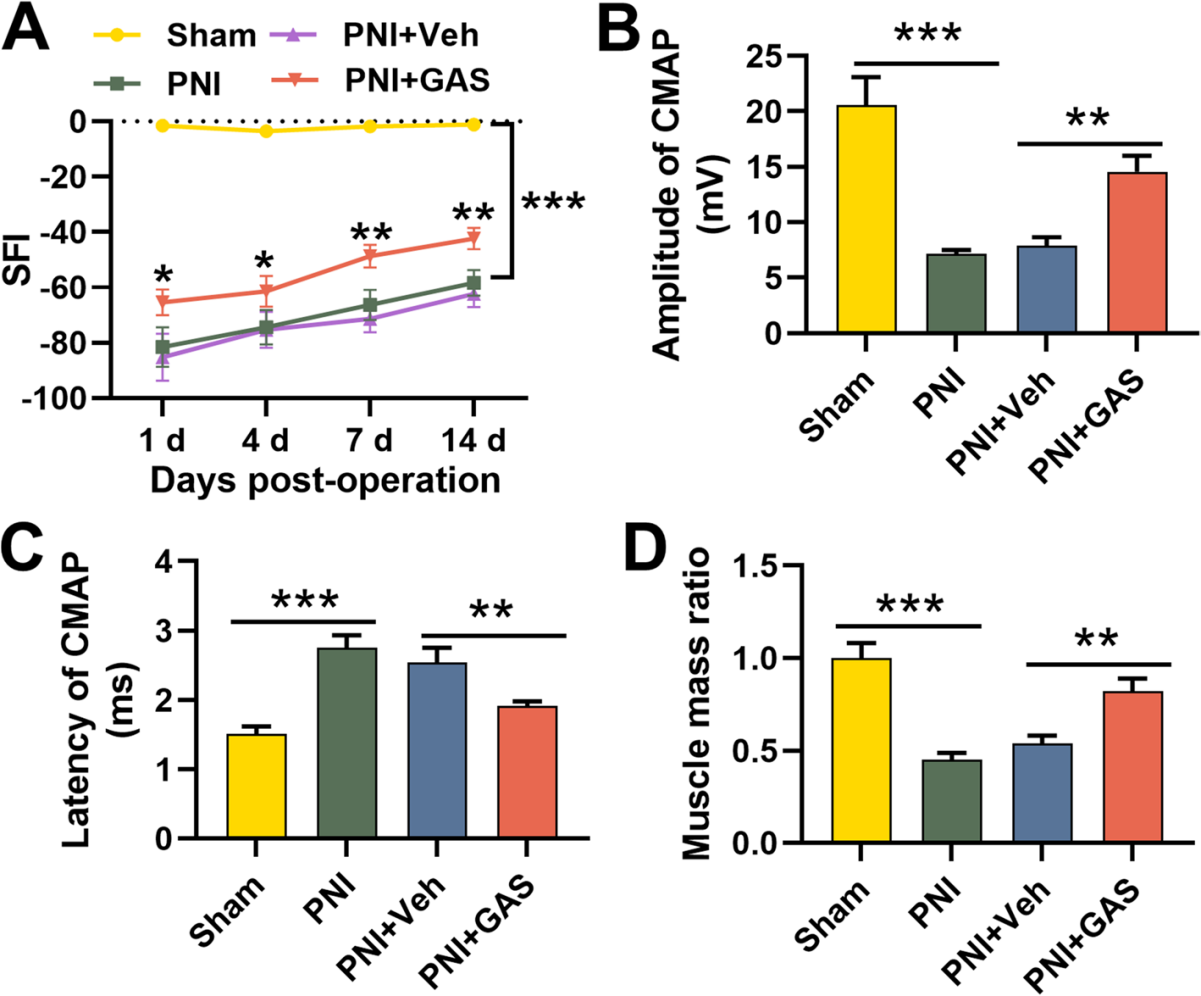
GAS promotes the recovery of neurological function of rats with PNI. A. SFI was used to determine the motor function of rats, and the results indicated that GAS treatment could promote the recovery of neurological function of the rats after PNI; **B**-**D**. GAS treatment significantly affected the amplitude (**B**), latency (**C**) of CMAP and gastrocnemius weight. Sham, sham operation group; PNI, peripheralnerve injury group; PNI + Veh, peripheralnerve injury and normal saline injection group; PNI + GAS, peripheralnerve injury and GAS treatment group. * *P* < 0.05, ** *P* < 0.01, and *** *P* < 0.001

First of all, we adopted SFI to evaluate the effect of GAS on motor function recovery of the rats with PNI and found that the SFI of the PNI group was markedly lower than that of the Sham group; the SFI of the GAS treatment group gradually increased, and it was higher than that of the PNI + Veh group (Fig. 1A). Moreover, GAS treatment increased CMAP amplitude and shortened the latency (Fig. 1B-C). Additionally, relative to the Sham group, the weight of the gastrocnemius muscle in the PNI group was markedly reduced, and GAS treatment partly reversed this effect, indicating that GAS observably ameliorated muscle atrophy (Fig. 1D). These results implied that GAS treatment could significantly promote nerve repair after PNI in vivo.

### GAS is beneficial to remyelination and nerve regeneration after PNI and relieves the oxidative stress response

**Fig. 2:**
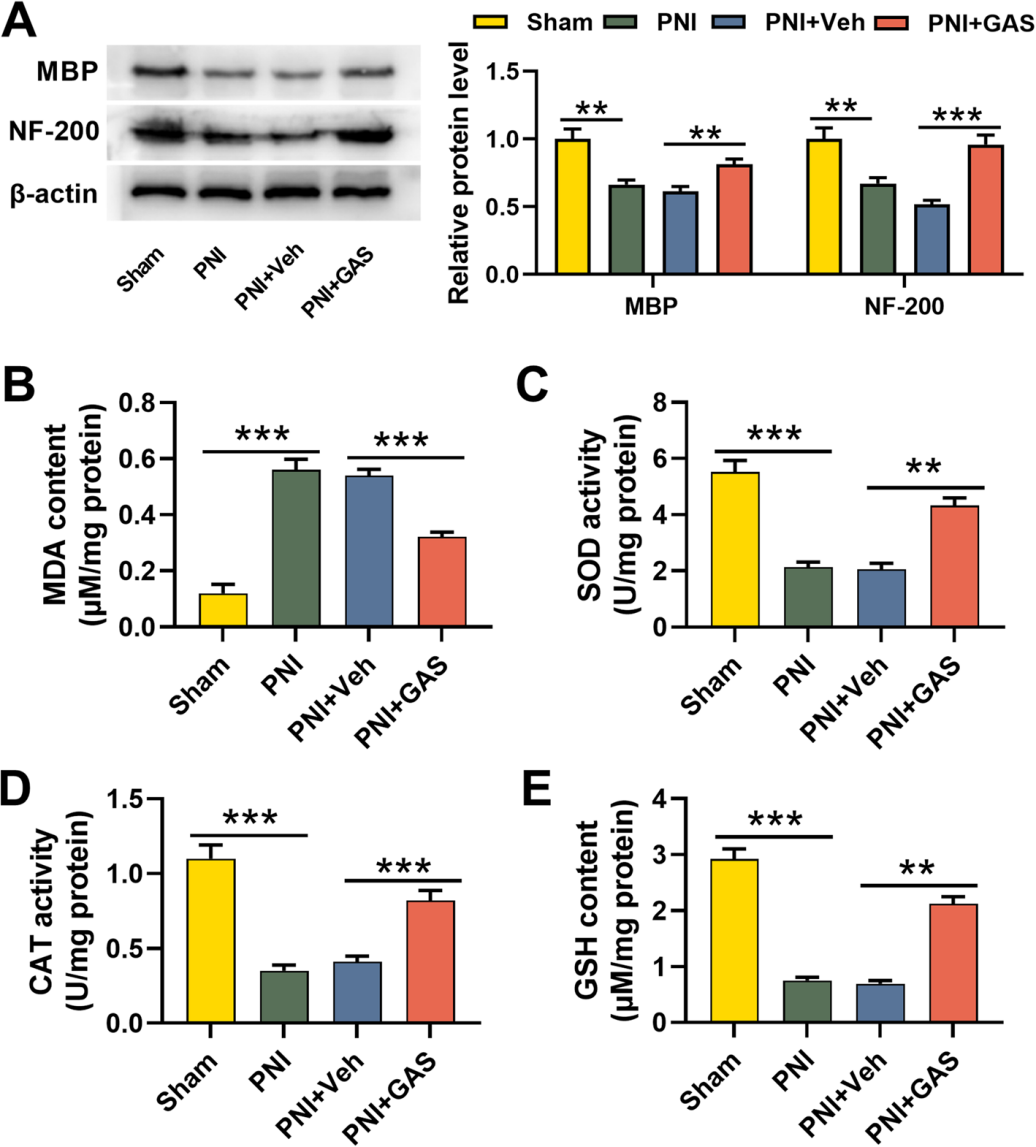
GAS is beneficial to the remyelination and nerve regeneration after PNI and relieves the oxidative stress response. **A**. Western blotting assay was adopted to detect MBP and NF-200 expressions in the sciatic nerve tissues of the rats in each group; **B**-**C**. The levels of oxidative stress indicators MDA, SOD, CAT, and GSH in the sciatic nerve tissues of the rats in each group were measured. Sham, sham operation group; PNI, peripheralnerve injury group; PNI + Veh, peripheralnerve injury and normal saline injection group; PNI + GAS, peripheralnerve injury and GAS treatment group. ** *P* < 0.01, and *** *P* < 0.001

To expound the impact of GAS on remyelination and nerve regeneration after PNI, we employed Western blotting assay to detect MBP and NF-200 expressions in the sciatic nerves after PNI, the findings of which showed that as against the Sham group, MBP and NF-200 expressions in the PNI group were reduced, implying that the structure and function of the myelin sheath were destroyed; compared with the PNI + Veh group, GAS treatment could increase MBP and NF-200 expressions, suggesting that GAS promoted the remyelination and nerve regeneration (Fig. 2A). It is reported that PNI is often accompanied by oxidative stress [31, 32]. Therefore, we assessed oxidative stress levels in the nerve tissue of the rats in each group via measuring the MDA content, SOD activity, CAT activity, and GSH content. In contrast to in the Sham group, the MDA content in the PNI group was increased significantly while that of the GAS treatment group was decreased (Fig. 2B). The SOD activity, CAT activity and GSH content in the PNI group were markedly lower than those in the Sham group, while those of GAS treatment group were observably increased compared with the PNI + Veh group (Fig. 2C-E). These data indicated that GAS can attenuate oxidative stress response after PNI.

### MiR-497 and BDNF expressions in PNI of rats

**Fig. 3:**
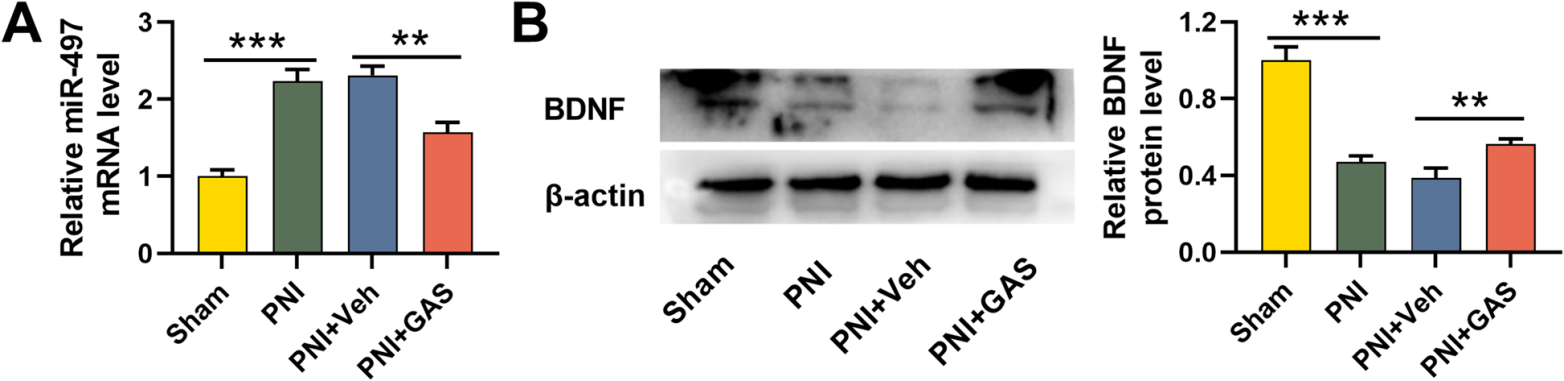
Expressions of miR-497 and BDNF in PNI of rats. **A**-**B**. qRT-PCR and Western blotting assay were adopted to detect the effect of GAS treatment on the expressions of miR-497 and BDNF in the sciatic nerve tissues of the rats in each group. Sham, sham operation group; PNI, peripheralnerve injury group; PNI + Veh, peripheralnerve injury and normal saline injection group; PNI + GAS, peripheralnerve injury and GAS treatment group. ** *P* < 0.01, and *** *P* < 0.001

Next, we examined miR-497 expression and BDNF protein expression in sciatic nerves of the rats by qRT-PCR and Western blotting assays, respectively. As shown, miR-497 expression was greatly increased and BDNF protein expression was markedly down-regulated in PNI group in comparison with those in the sham group; however, as against the PNI + Veh group, miR-497 expression was remarkably declined and BDNF protein expression was significantly elevated after GAS treatment (Fig. 3A-B). These results manifested that GAS could inhibit miR-497 expression and facilitate BDNF expression to participate in the recovery of peripheral nerve after PNI.

### BDNF is the target of miR-497

**Fig. 4:**
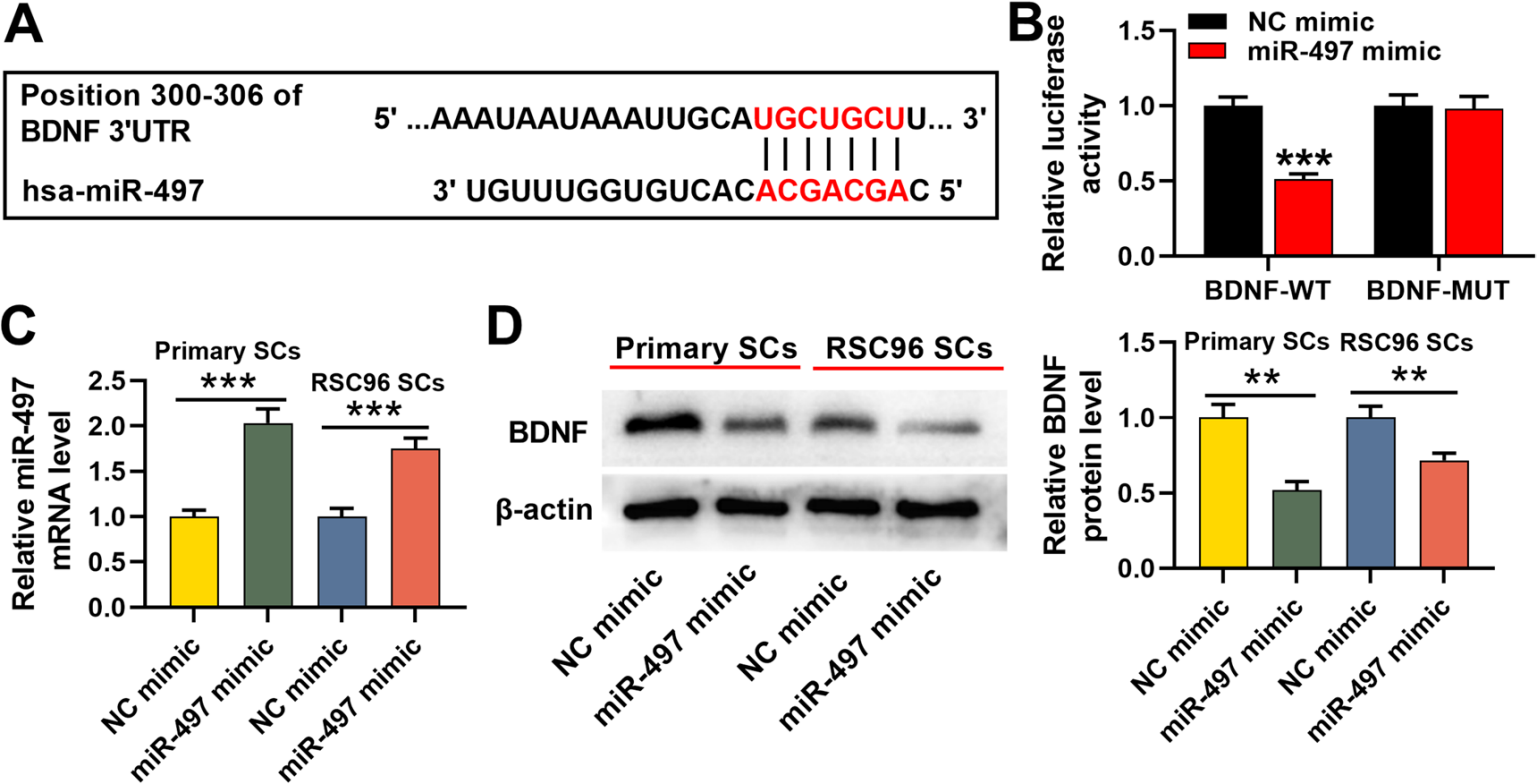
BDNF is the target of miR-497. **A**. StarBase and TargetScan databases were employed to predict the target site sequences between miR-497 and BDNF; **B**. Dual-luciferase reporter gene assay was used to verify the targeting relationship between miR-497 and BDNF; **C**-**D**. MiR-497 mimic and NC mimic were transfected into primary SCs and RSC96 SCs, respectively, and the relative expressions of miR-479 and BDNF proteins were detected by qRT-PCR and Western blotting assay. ** *P* < 0.01, and *** *P* < 0.001

As reported, BDNF is a target of miR-497, and miR-497/BDNF axis regulates the neural injury induced by anesthesia [33]. Additionally, StarBase and TargetScan databases also predicted the existence of a target site between miR-497 and BDNF 3’UTR (Fig. 4A). We then explored whether miR-497 could regulate BDNF expression in SCs. Dual-luciferase reporter gene assay indicated that, when BDNF-WT reporter and miR-497 mimics were accordingly co-transfected into HEK293T cells, the relative activity of luciferase was markedly inhibited, but that of BDNF-MUT reporter was not significantly altered by miR-497 mimics (Fig. 4B). Then we transfected miR-497 mimics or NC mimics into SCs (Primary SCs and RSC96 SCs). As shown, miR-497 mimics observably increased miR-497 expression (Fig. 4C); moreover, the overexpression of miR-497 mimics markedly inhibited BDNF expressions in SCs (Fig. 4D).

### MiR-497 modulates the migration and viability of SCs through BDNF

**Fig. 5:**
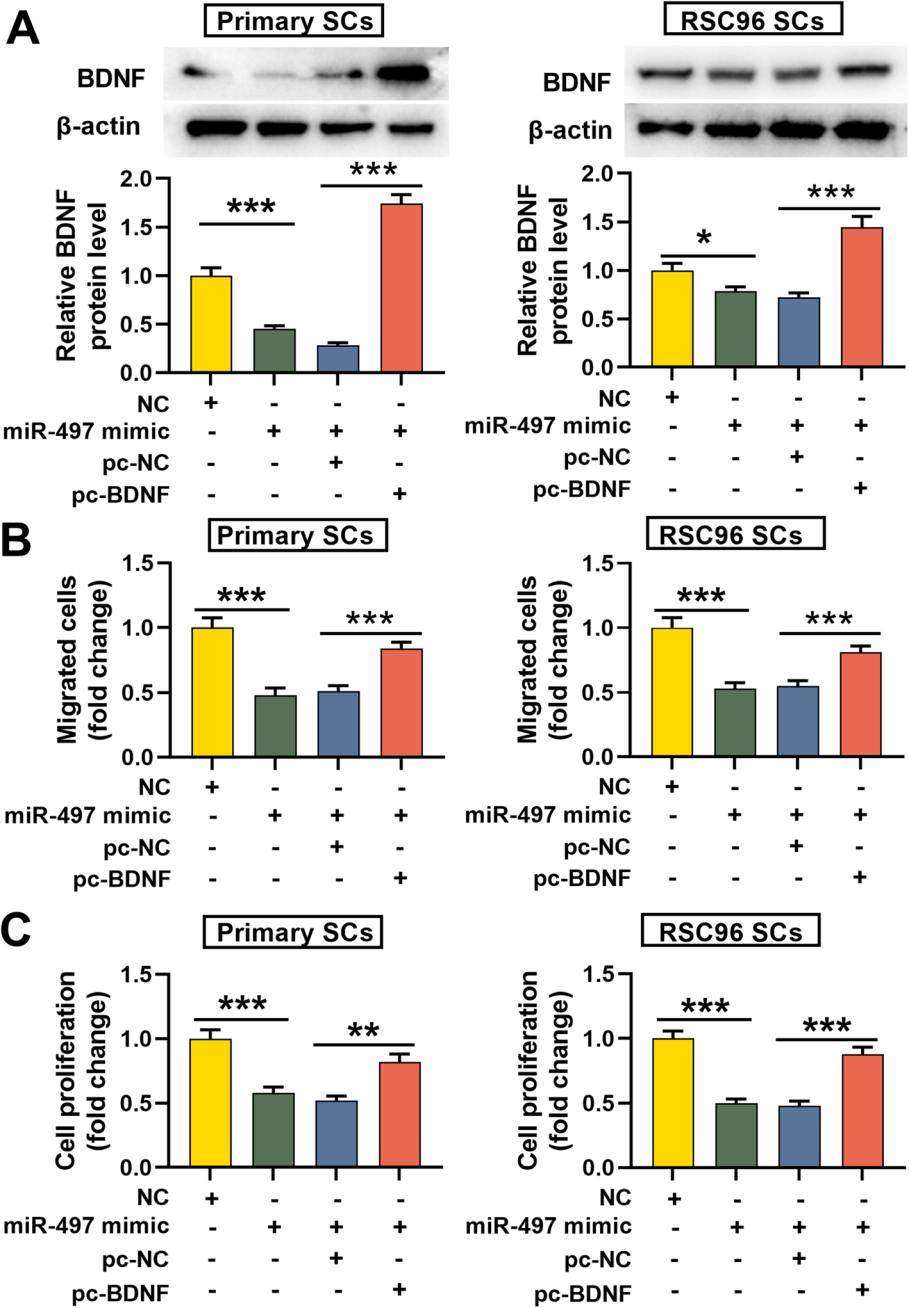
MiR-497 regulates the migration and proliferation of SCs through BDNF. **A**. BDNF overexpression plasmid was co-transfected into SCs with miR-497 overexpression, and the relative protein expression of BDNF was detected by Western blotting assay; **B**-**C**. The effects of miR-497 and BDNF on the migration and proliferation of SCs were examined by Transwell and EdU assays, respectively. ** *P* < 0.01, and *** *P* < 0.001

To fathom how miR-497 modulates the migration and viability of SCs through BDNF, we transfected pc-BDNF or pc-NC into SCs with miR-497 overexpression. Western blotting assay showed that the transfection of pc-BDNF significantly restored BDNF expression (Fig. 5A). Functional experiments revealed that in SCs transfected with miR-497 mimic, the migration and proliferation of SCs were remarkably reduced, while BDNF restoration reversed this effect (Fig. 5B-C). These findings proved that miR-497 could repress the migration and viability of SCs via repressing BDNF expression.

### GAS reduces the oxidative stress response of SCs

**Fig. 6:**
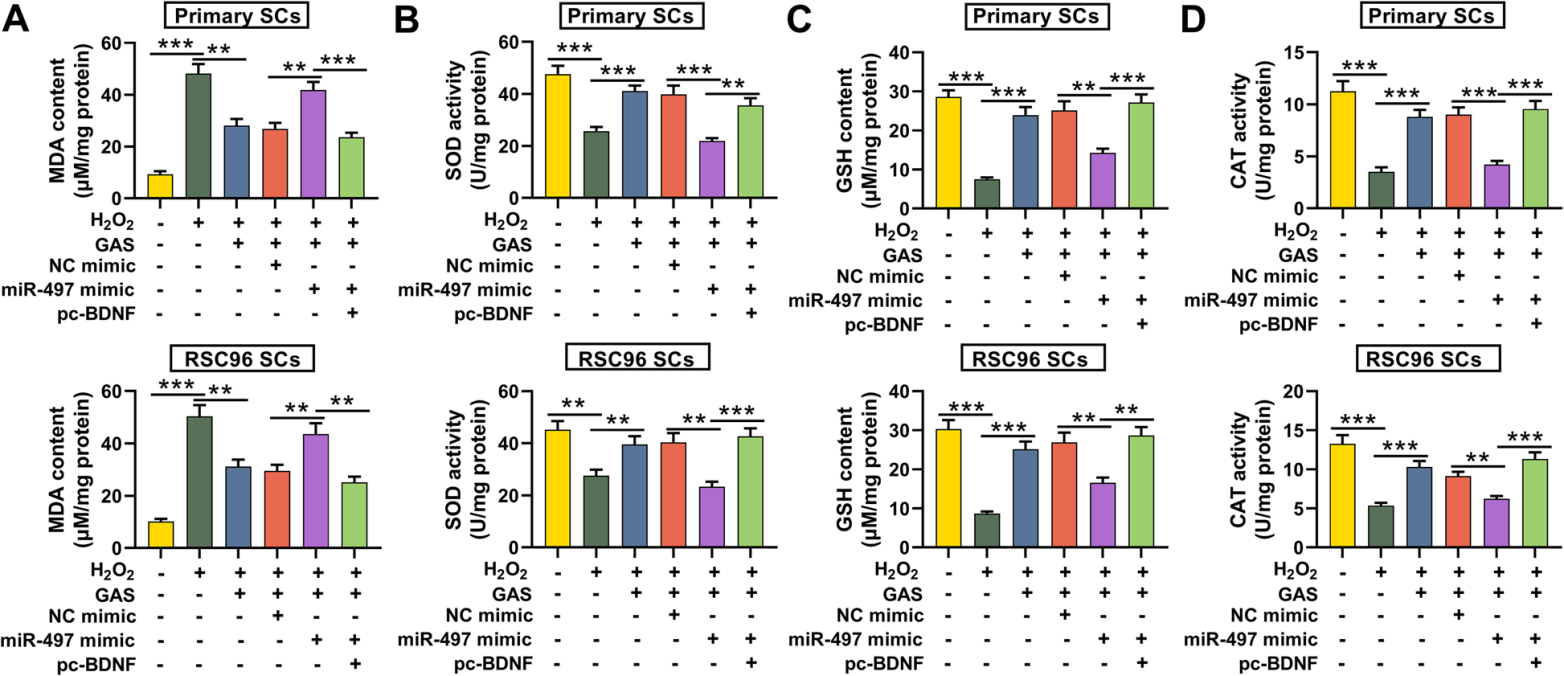
GAS reduces the oxidative stress response of SCs induced by H_2_O_2_ via regulating miR-497/BDNF axis. **A**-**D**. The changes in MDA content, SOD activity, GSH content and CAT activity in H_2_O_2_-treated SCs were detected. ** *P* < 0.01, and *** *P* < 0.001

H_2_O_2_-induced oxidative damage was established with SCs. MDA, SOD, GDH and CAT levels/activities in SCs were measured to further explore the effect of GAS on oxidative stress and the results implied that the MDA content in H_2_O_2_-treated SCs was markedly higher than that in the control group, while the SOD, CAT activities and GSH levels were lower than those in the control group; however, GAS treatment markedly repressed MDA level in SCs but promoted SOD activity, CAT activity and GSH level; furthermore, the transfection of miR-497 mimics abolished the impact of GAS on the oxidative stress of SCs; additionally, BDNF restoration counteracted the functions of miR-497 (Fig. 6A-D). These results highlighted that GAS alleviated the oxidative stress response of SCs through regulating miR-497/BDNF pathway.

## Discussion

Despite the prevalence of PNI, there is no effective treatment strategy. In recent years, various drugs have been tried to promote the axon and nerve regeneration to treat nerve injury in many preclinical studies [34–38]. For example, vitamin B12 is used for the neuroprotection of peripheral neuropathy [35]. Ginsenoside Rg1 promotes the anti-oxidation and nerve regeneration in a rat model of PNI [36, 37]. Isoquercitrin may facilitate nerve regeneration and functional recovery via inhibiting oxidative stress [38]. GAS has been considered as a promising drug for neuroprotection. Specifically, GAS treatment could promote neurogenesis and ameliorates ischemic injury in a mice model with stroke [39]. Additionally, GAS improves the neural tissue recovery in the injured spinal cord in rats [40]. In this study, with a rat model with PNI, we proved that GAS treatment markedly ameliorated PNI-induced muscle atrophy, increased CMAP amplitude, and reduced refractory period. This implied that GAS was a promising drug to treat PNI by improving nerve regeneration and functional recovery.

It is well known that the regeneration of myelin sheath and nerve is essential for the reconstruction of functional nerves that innervate their corresponding target tissues [41]. MBP is the main component of the myelin sheath produced by the SCs in PNS; and NF200 is a marker of myelinated neurons and is essential for the stabilization of axons during maturation [42]. In this work, it was demonstrated that GAS treatment markedly increased MBP and NF-200 expressions to promote nerve regeneration and myelination. Besides, oxidative stress is one of the main pathological factors contributing to neuronal injury, and it has a negative effect on peripheral nerve regeneration and the recovery of neurological function after PNI [43–45]. Accumulating studies elucidate that inhibiting post-PNI oxidative stress can expedite the repair process and promote functional recovery after nerve injury [46, 47]. GAS is reported to have significant antioxidant activities [48, 49]. For instance, it is reported that GAS has neuroprotective effects on early brain injury after subarachnoid hemorrhage by significantly reducing the oxidative stress [49]. In the present work, our data showed that GAS could promote SOD activity, CAT activity and GSH content in nerve tissue of rats treated with PNI, and decrease MDA content. These data suggested that the protective function of GAS on peripheral nerve was dependent on its role in promoting neuroregeneration and repressing oxidative stress response.

MiRNAs take part in regulating neurodevelopment and the pathogenesis of neuronal injury [50]. For example, let-7 miRNAs participate in the repair processes of sciatic nerve by regulating myelination [51]. Reportedly, SCs synthesize and secrete neurotrophic factors during peripheral nerve regeneration, including nerve growth factor, BDNF, neurotrophin-3/4/5, thereby enhancing the survival and growth of neurons [3]. Reported, GAS can not only promote the secretion of BDNF in rats with spinal cord injury, but also up regulate BDNF expressions in hippocampus and hippocampal astrocytes [25, 40]. MiRNA can regulate the survival and migration of SCs to regulate the recovery of PNI. For instance, miR-182 expression is up-regulated after sciatic nerve injury in rats and the overexpression of miR-182 represses the proliferation and migration of SCs via repressing fibroblast growth factor 9 and neurotrimin, contributing to the injury of neurons [52]. The present study unmasked that miR-497 and BDNF expression were dysregulated in the sciatic nerve tissues of rats with PNI, and GAS markedly suppressed miR-497 expression and up-regulated BDNF expression. We further verified the targeting relationship between miR-497 and BDNF in SCs. Our data also confirmed that miR-497 overexpression significantly restrained the proliferation and migration of SCs, while BDNF overexpression reversed this effect. Therefore, we concluded that GAS could probably be beneficial to axon growth and myelination via modulating the miR-497/BDNF axis to promote the growth and migration of SCs cells.

## Conclusion

To recapitulate briefly, this work reveals that GAS regulates the miR-497/BDNF axis to facilitate the migration and proliferation of SCs, inhibit oxidative stress and thereby accelerate myelination, nerve regeneration and functional recovery after PNI. These data help clarify the mechanism of PNI’s pathogenesis, and explain the molecular mechanism of the neuroprotective function of GAS. Our study provides a theoretical basis for the application of GAS and GEB in clinical treatment of PNI.

## Supplementary Information

**Additional file 1.** The original images of Western blot assay.

## Data Availability

The data used to support the findings of this study are available from the corresponding author upon request.

## Abbreviations

GAS: Gastrodin
DCXF: Da Chuanxiong Formula
BBB: Blood-brain barrier
PNI: Peripheral nerve injury
SFI: Sciatic nerve function index
CMAP: Compound muscle action potential
SCs: Schwann cells
BDNF: Brain-derived neurotrophic factor
miRNA: microRNA
NF-200: Neurofilament-200
MBP: Myelin basic protein
MDA: Malondialdehyde
SOD: Superoxide dismutase
CAT: Catalase
GSH: Glutathione
CNS: Central nervous system
PNS: Peripheral nervous system
NGF: Nerve growth factor
GEB: Gastrodia elata Blume
