# Supplementary material for: Gastrodin promotes the regeneration of peripheral nerves by regulating miR-497/BDNF axis
Source: BMC Complement Med Ther. 2022 Feb 18;22:45. doi: 10.1186/s12906-021-03483-z (PMC8855574; doi:10.1186/s12906-021-03483-z)

**Supplementary Materials 1 The original picture of Western blot assay.**

**Figure 2A MBP**


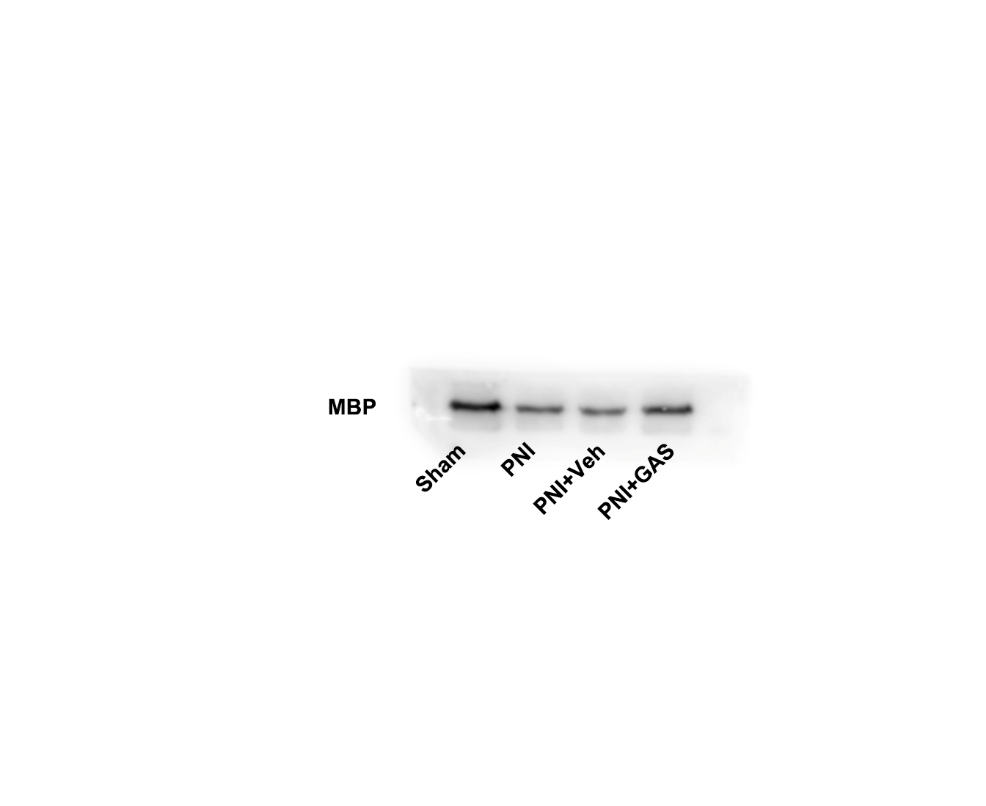


**Figure 2A NF-200**


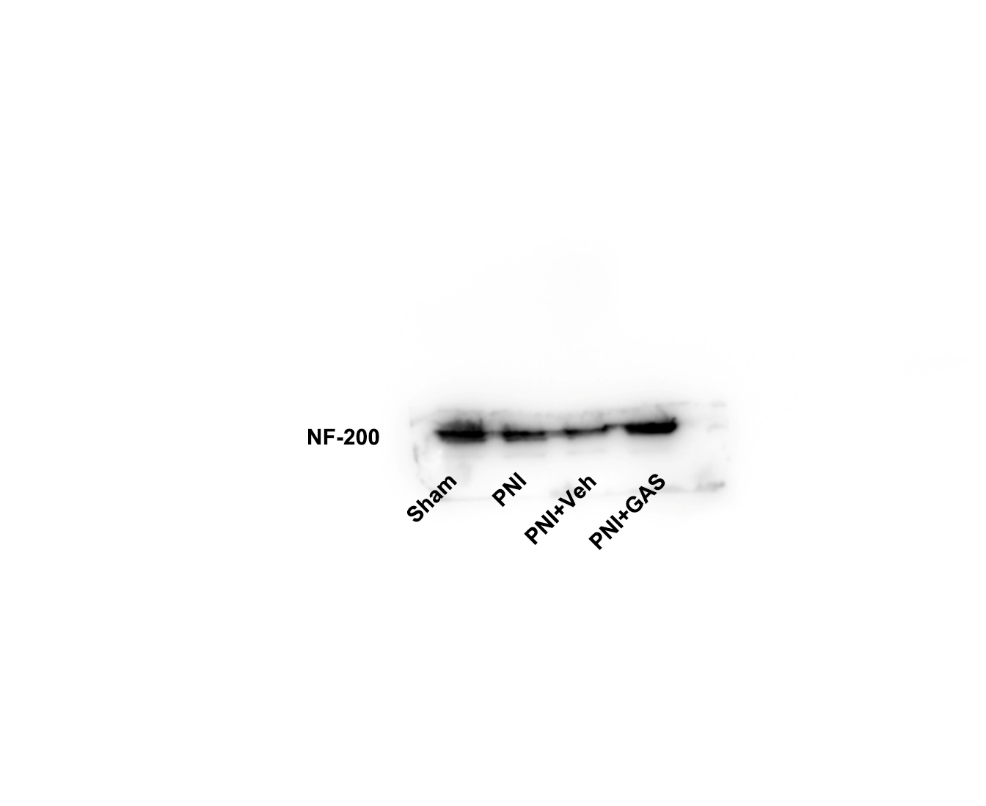


**Figure 2A β-actin**


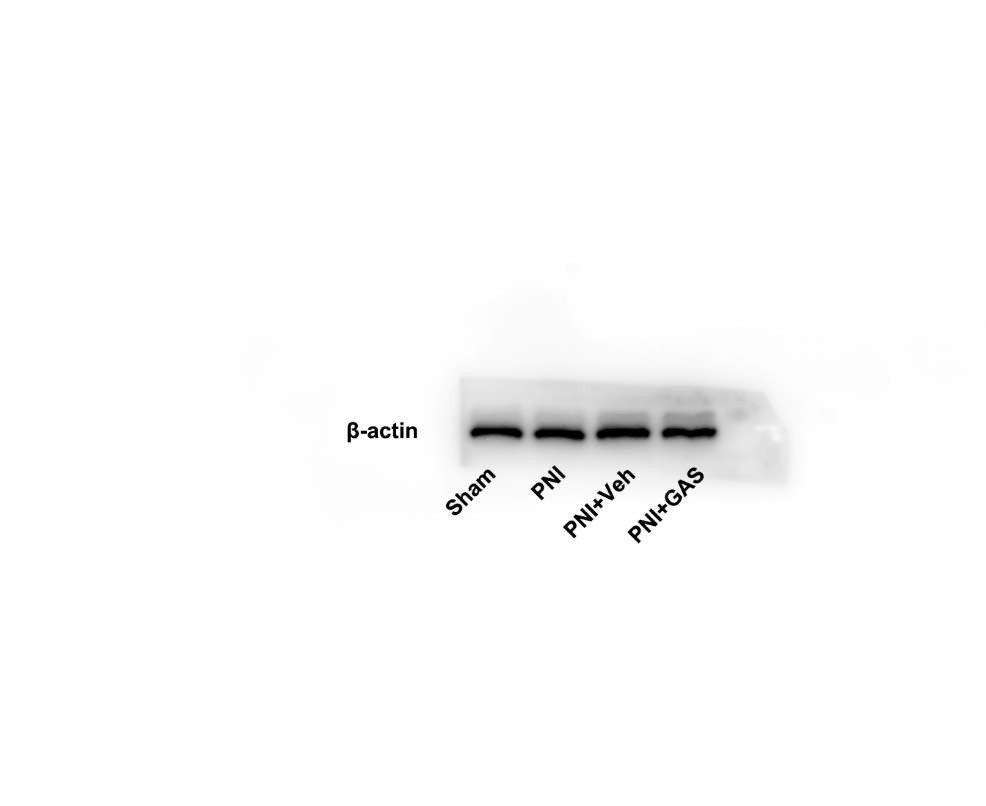


**Figure 3B BDNF**


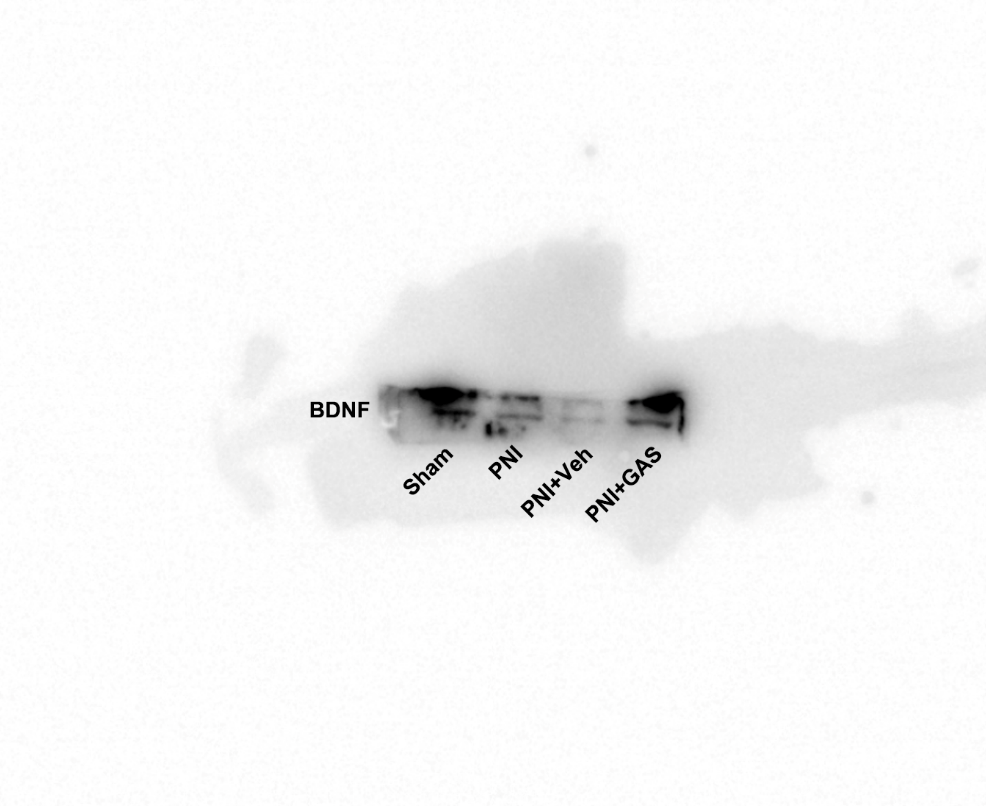


**Figure 3B β-actin**


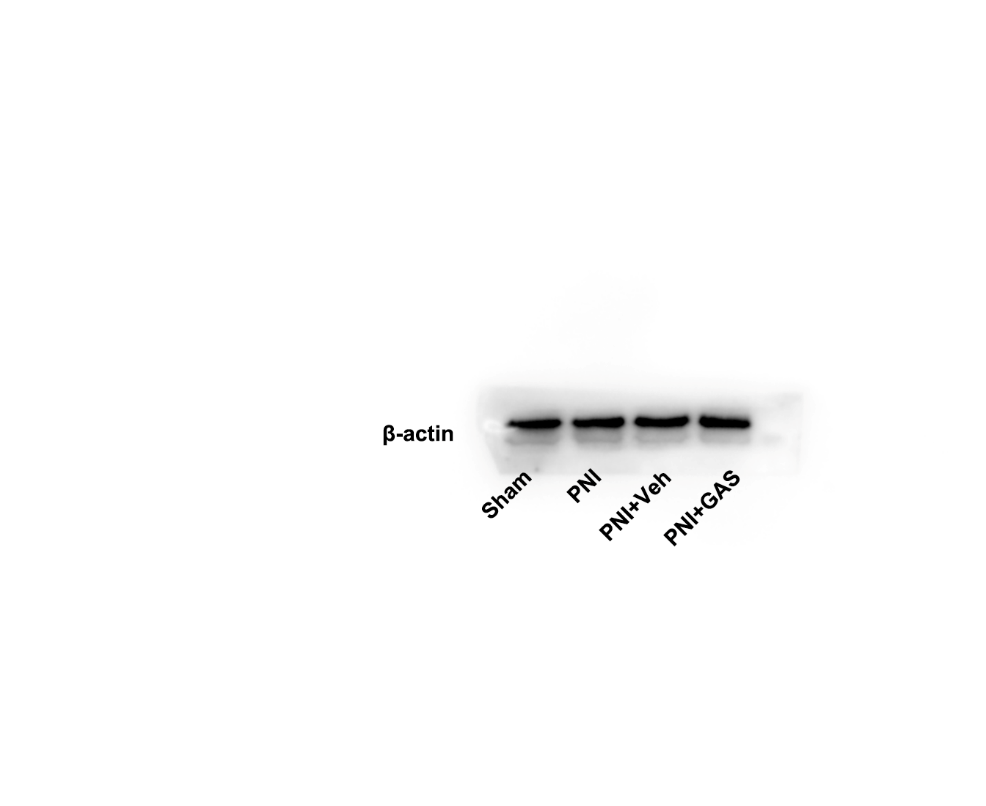


**Figure 4D BDNF**


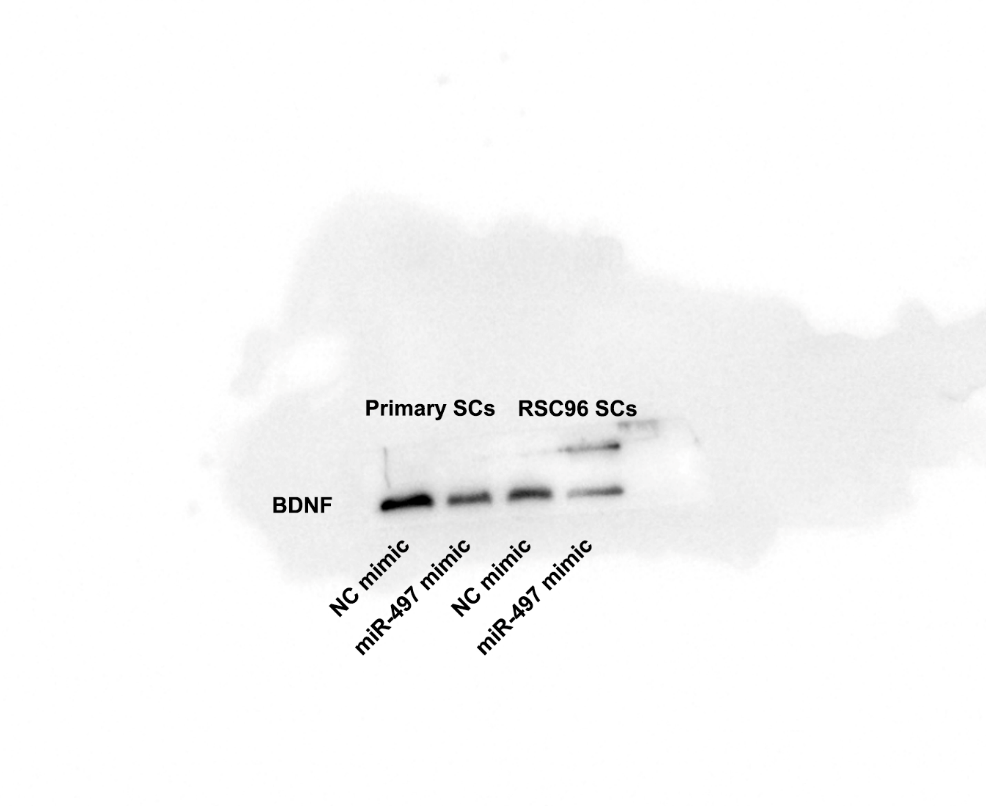


**Figure 4D β-actin**


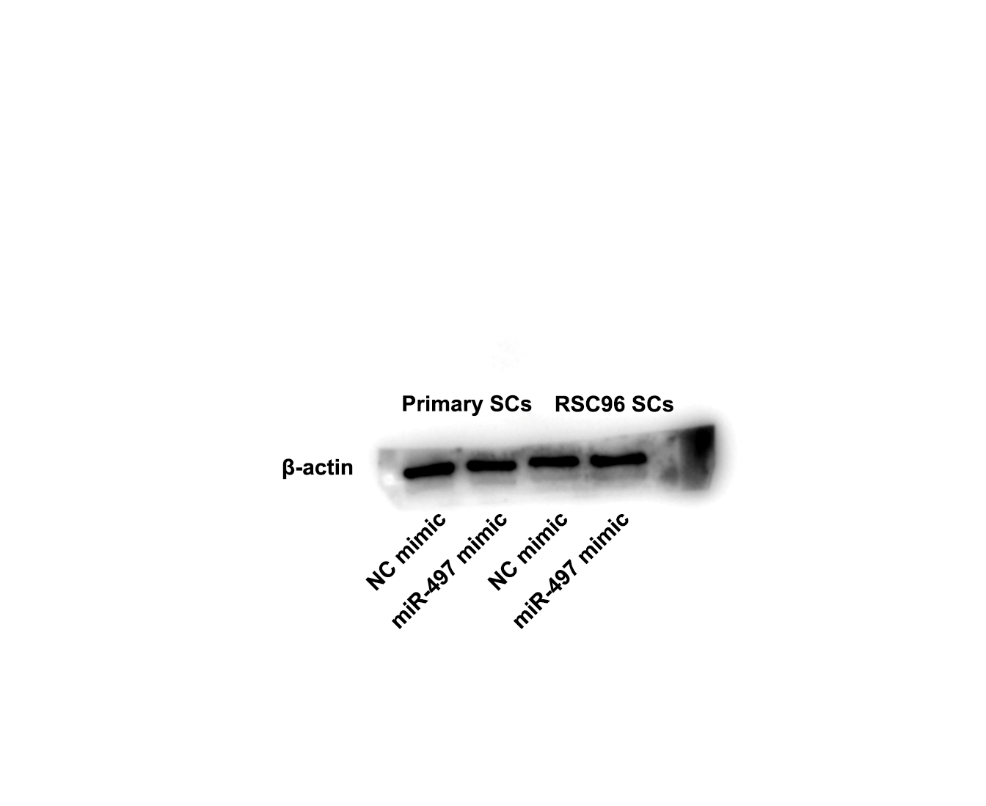


**Figure 5A Primary SCs BDNF**


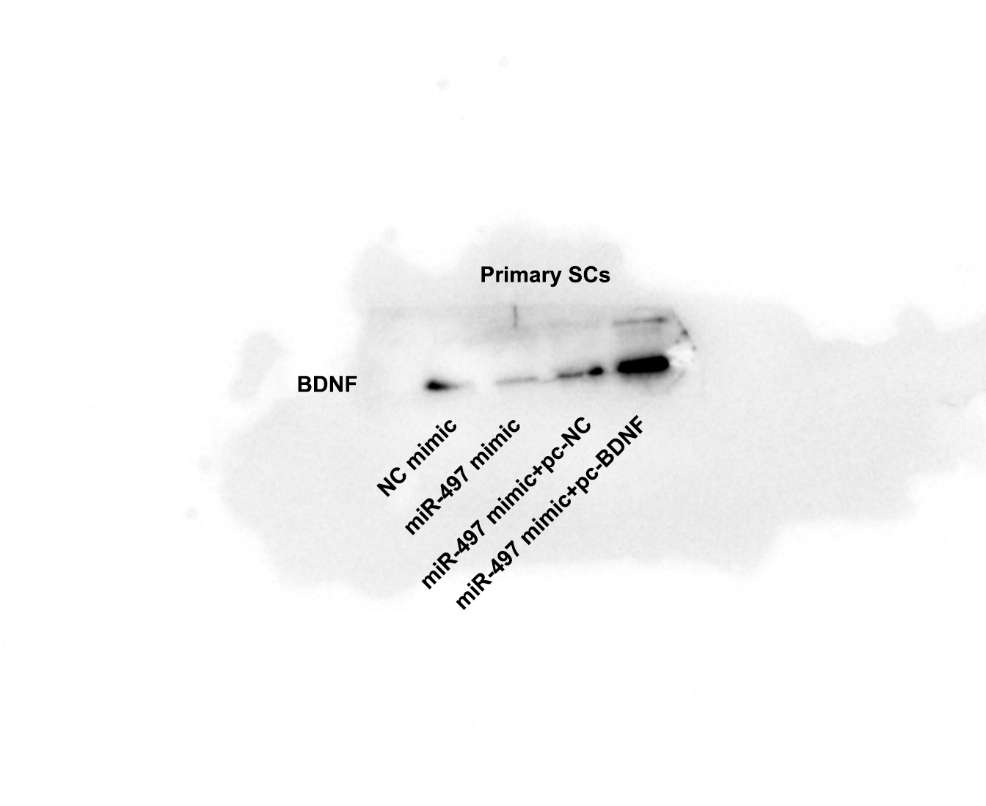


**Figure 5A Primary SCs β-actin**


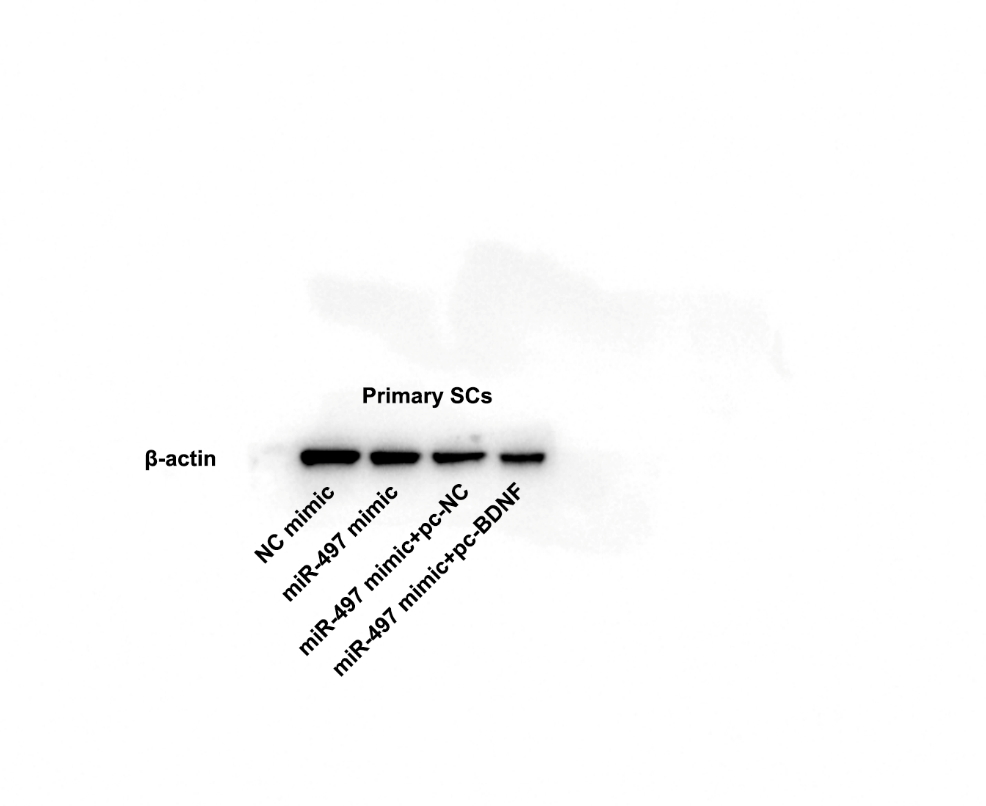


**Figure 5A RSC96 SCs BDNF**


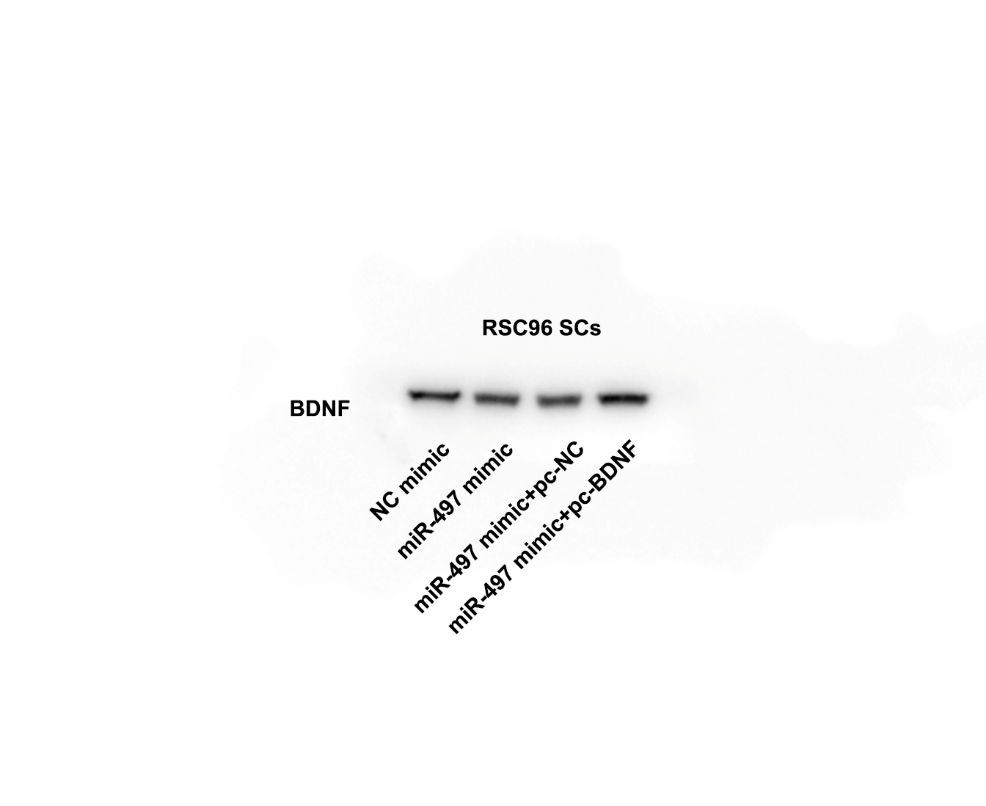


**Figure 5A RSC96 SCs β-actin**


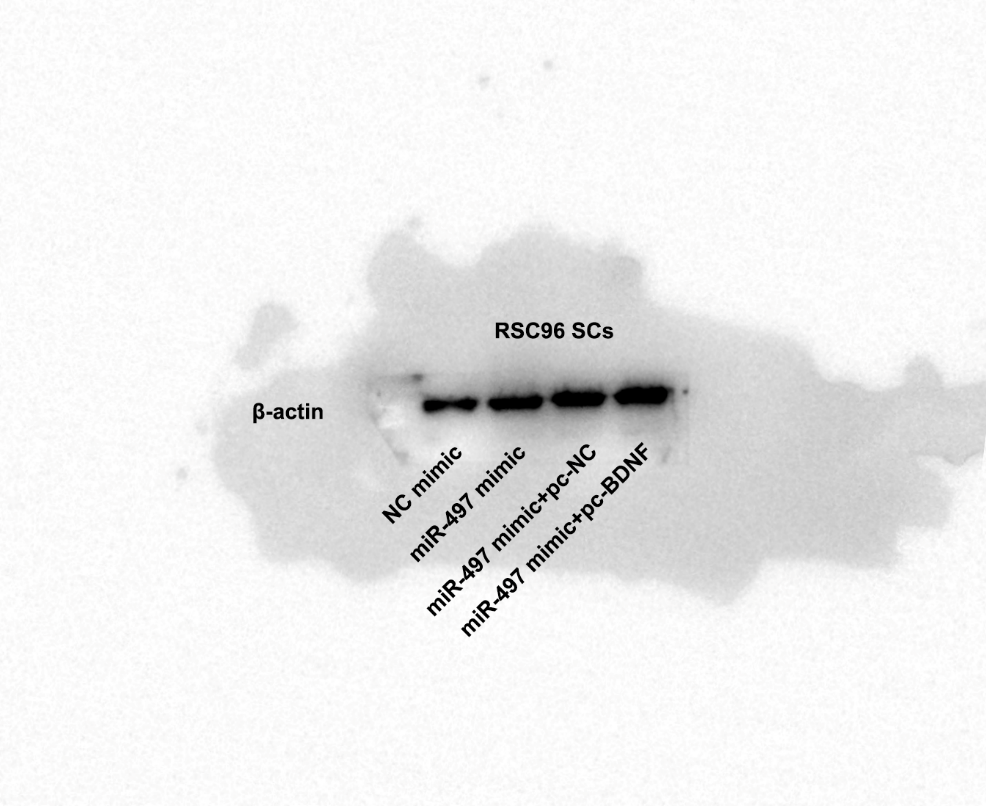

Supplement: Supplementary file 1 — Additional file 1. The original images of Western blot assay. [file 12906_2021_3483_MOESM1_ESM.docx]
